# Middle cerebral arterial flow redistribution is an indicator for intrauterine fetal compromise in late pregnancy in low‐resource settings: A prospective cohort study

**DOI:** 10.1111/1471-0528.17115

**Published:** 2022-02-24

**Authors:** Sam Ali, Michael G. Kawooya, Josaphat Byamugisha, Isaac M. Kakibogo, Esther A. Biira, Adia N. Kagimu, Diederick E. Grobbee, David Zakus, Aris T. Papageorghiou, Kerstin Klipstein‐Grobusch, Marcus J. Rijken

**Affiliations:** ^1^ Julius Global Health, Julius Center for Health Sciences and Primary Care, University Medical Center Utrecht Utrecht University Utrecht The Netherlands; ^2^ School of Medicine Makerere University College of Health Sciences Kampala Uganda; ^3^ Ernest Cook Ultrasound Research and Education Institute (ECUREI), Mengo Hospital Kampala Uganda; ^4^ Antenatal and Maternity Unit, Kagadi Hospital Kagadi District Uganda; ^5^ The Women's Place Kampala Uganda; ^6^ Division of Clinical Public Health, Dalla Lana School of Public Health University of Toronto Toronto Ontario Canada; ^7^ Nuffield Department of Women’s and Reproductive Health John Radcliffe Hospital, University of Oxford Oxford UK; ^8^ Division of Epidemiology and Biostatistics, School of Public Health, Faculty of Health Sciences University of the Witwatersrand Johannesburg South Africa; ^9^ Department of Obstetrics and Gynecology, University Medical Center Utrecht Utrecht University Utrecht the Netherlands

**Keywords:** cerebroplacental ratio, developing countries, Doppler ultrasound, perinatal death, prediction, prenatal care, screening, stillbirth

## Abstract

**Objective:**

We aimed to determine the prevalence of abnormal umbilical artery (UA), uterine artery (UtA), middle cerebral artery (MCA) and cerebroplacental ratio (CPR) Doppler, and their relationship with adverse perinatal outcomes in women undergoing routine antenatal care in the third trimester.

**Design:**

Prospective cohort.

**Setting:**

Kagadi Hospital, Uganda.

**Population:**

Non‐anomalous singleton pregnancies.

**Methods:**

Women underwent an early dating ultrasound and a third‐trimester Doppler scan between 32 and 40 weeks of gestation, from 2018 to 2020. We handled missing data using multiple imputation and analysed the data using descriptive methods and a binary logistic regression model.

**Main outcome measures:**

Composite adverse perinatal outcome (CAPO), perinatal death and stillbirth.

**Results:**

We included 995 women. The mean gestational age at Doppler scan was 36.9 weeks (SD 1.02 weeks) and 88.9% of the women gave birth in a health facility. About 4.4% and 5.6% of the UA pulsatility index (PI) and UtA PI were above the 95th percentile, whereas 16.4% and 10.4% of the MCA PI and CPR were below the fifth percentile, respectively. Low CPR was strongly associated with stillbirth (OR 4.82, 95% CI 1.09–21.30). CPR and MCA PI below the fifth percentile were independently associated with CAPO; the association with MCA PI was stronger in small‐for‐gestational‐age neonates (OR 3.75, 95% CI 1.18–11.88).

**Conclusion:**

In late gestation, abnormal UA PI was rare. Fetuses with cerebral blood flow redistribution were at increased risk of stillbirth and perinatal complications. Further studies examining the predictive accuracy and effectiveness of antenatal Doppler ultrasound screening in reducing the risk of perinatal deaths in low‐ and middle‐income countries are warranted.

**Tweetable abstract:**

Blood flow redistribution to the fetal brain is strongly associated with stillbirths in low‐resource settings.

## Tweetable abstract

Blood flow redistribution to the fetal brain is strongly associated with stillbirths in low‐resource settings.

This article includes Author Insights, a video abstract available at https://vimeo.com/bjogabstracts/authorinsights17115.

## 1 INTRODUCTION

Of the nearly 7000 stillbirths daily worldwide, three‐quarters occur in South Asia and sub‐Saharan Africa.^1^ About half are antepartum stillbirths, frequently associated with fetal growth restriction (FGR), secondary to placental insufficiency.^1^ In low‐ and middle‐income countries (LMICs), one in five infants is born small for gestational age (SGA) and 22% of neonatal deaths are among such infants.^2^

Growth‐restricted fetuses are not only at high risk of perinatal death but are also at risk of short‐ and long‐term complications, making detection and management crucial.^2^, ^3^, ^4^ However, their prenatal detection rates are low, particularly at late gestation.^2^ According to data from high‐income countries (HICs), late‐onset FGR is usually mild in presentation and often associated with normal umbilical and uterine arterial Doppler indices.^5^, ^6^, ^7^, ^8^ Nevertheless, abnormal fetal cerebral Doppler flow is a frequent finding in such fetuses.^7^, ^8^ There is still limited evidence on the clinical value of a third‐trimester Doppler ultrasound for fetal surveillance in low‐risk pregnancies or women undergoing routine antenatal care (ANC), particularly in LMICs.^9^ The World Health Organization (WHO) recommends well‐conducted studies to provide concrete evidence for its use in ANC.^9^

Some studies, mostly from HICs, show significant correlations between abnormal umbilical and middle cerebral artery Doppler measurements and perinatal complications in fetuses that appear appropriately grown upon ultrasound.^10^, ^11^, ^12^, ^13^, ^14^, ^15^ This suggests a potential role for Doppler ultrasound in preventing stillbirths among women with low‐risk pregnancies or undergoing routine ANC. Although its effectiveness as a screening tool has not been established in these HIC settings, it is clear that test performance depends on the underlying prevalence of perinatal complications or abnormal Doppler in the general population.^16^ This is of relevance to LMIC settings where stillbirths are ten times more common than in HICs.^1^ It should also be noted that, because of a lack of studies, a recent systematic review was unable to establish the prevalence in LMIC settings of absent or reversed end‐diastolic flow (AEDF or REDF) in the umbilical artery (UA) among low‐risk pregnancies or women undergoing routine ANC.^17^ The finding was rare (0%–2%) in HICs.^17^ Therefore, this study aimed to investigate the prevalence of abnormal antenatal Doppler ultrasound findings and their relationship with adverse perinatal outcomes in a cohort of women undergoing routine ANC during the third trimester in Uganda.

## 2 METHODS

### 2.1 Study design

This prospective cohort study was implemented between September 2018 and December 2020 at Kagadi Hospital, a secondary healthcare facility serving as a referral centre for the neighbouring districts in the greater Kibaale region in mid‐western Uganda. According to the District Health Information System (DHIS), less than 10% of women present to Kagadi Hospital for their first ANC contact in the first trimester annually. In 2020, 264/3080 women presented for their first ANC in the first trimester, 1483 achieved four or more ANC contacts and only 13 achieved the recommended eight ANC contacts. The hospital handles over 4000 births a year. This study recruited women from the ANC clinic, maternity unit and ultrasound department.

### 2.2 Eligibility criteria

We included women pregnant with singletons who enrolled in early pregnancy (<24 weeks of gestation), with no obvious fetal abnormalities on the scan. The exclusion criteria included women with missed miscarriage or intrauterine fetal demise, and birth before the Doppler scan (i.e. those who gave birth before 33 ^+ 0^ weeks of gestation). Gestational age (GA) was confirmed by a first‐trimester ultrasound between 9 ^+ 0^ and 13 ^+ 6^ weeks of gestation using the crown–rump length (CRL)^18^ or head circumference combined with femoral length measurements taken before 24 weeks of getsation.^19^

### 2.3 Data collection

The first author (SA) and two resident sonographers at Kagadi Hospital carried out all the ultrasound examinations. The sonographers had experience performing obstetric ultrasound and they received additional training on fetal Doppler ultrasound at the Ernest Cook Ultrasound Research and Education Institute (ECUREI), Kampala, Uganda, and The Women’s Place, Kampala, Uganda, before beginning study implementation. Ultrasound examinations were performed using a Voluson™ e (GE Healthcare, Chicago, IL, USA) with a 2–8 MHz convex probe or Philips HD‐9 (Philips, Amsterdam, the Netherlands) equipped with C5‐2 and C6‐3 convex probes.

We performed all ultrasound scans according to standard protocols.^20^ Doppler scans were carried out between 32 and 40 weeks of gestation. The UA was examined in a free loop of the umbilical cord and measured in the absence of fetal movement, while keeping the insonation angle at <30°. The middle cerebral artery (MCA) was examined at its proximal third, close to its origin in the internal carotid artery, with the angle of insonation kept as close as possible to 0°. We recorded the uterine artery (UtA) Doppler trans‐abdominally, with the angle of insonation maintained at <30°. The mean value of the left and right UtA pulsatility index (PI) was calculated. All Doppler waveform velocities were recorded as the average of three similar consecutive waveforms. At the time of study, there were no local guidelines for the management of suspected SGA fetuses, and Doppler was not routinely used to manage pregnancies in the study setting. Therefore, clinicians were blinded to the Doppler results except when AEDF or REDF in the UA was detected. Any woman with an abnormal ultrasound finding was referred to the clinicians for management following the standard of care that the clinician deemed suitable. All women enrolled in the study were followed up until 28 days postnatally. Given the occurrence of COVID‐19 lockdowns we remotely captured some follow‐up data with phone calls, such as reports on neonatal death.

### 2.4 Maternal and pregnancy characteristics

The characteristics recorded included maternal age, weight and height, parity, malaria in pregnancy, urinary tract infection, syphilis, HIV status, previous stillbirth, chronic hypertension, alcohol use during pregnancy, maternal smoking, and gestational age at Doppler scan and birth. Ultrasound measurements included estimated fetal weight (EFW), abdominal circumference (AC), UA PI, MCA PI, cerebroplacental ratio (CPR) and mean UtA PI.

### 2.5 Outcome measures

The primary outcomes included a composite adverse perinatal outcome (CAPO), perinatal death and stillbirth. The CAPO was defined as the occurrence of one or more of the following: stillbirths (intrauterine fetal death after 28 weeks of gestation), neonatal death within 28 days of the postnatal period, admission to neonatal intensive care unit (NICU) for >24 hours, Apgar score of <7 at 5 minutes, emergency caesarean birth for fetal distress (based on abnormal fetal heart rate monitoring) and respiratory distress syndrome (RDS). Perinatal death was defined as the occurrence of either stillbirth or neonatal death within 28 days of the postnatal period. We defined SGA as birthweight <10th percentile and AGA as birthweight ≥10th percentile using the International Fetal and Newborn Growth Consortium for the 21st Century (INTERGROWTH‐21^st^) newborn birthweight standards.^21^

### 2.6 Statistical analysis

We computed EFW using Hadlock equation 3 and converted it to gestational‐specific *z*‐scores using the INTERGROWTH‐21^st^ charts.^22^, ^23^ We also converted fetal biometry and birthweight to gestational‐specific *z*‐scores and percentiles using the INTERGROWTH‐21^st^ fetal growth standards,^24^ and newborn birthweight standards after adjustment for sex.^21^ CPR was calculated as a ratio of the MCA PI to the UA PI. We transformed UA PI to gestational age‐specific *z*‐scores and percentiles using the INTERGROWTH‐21^st^ Doppler charts,^25^ and dichotomised it at the 95th percentile to estimate prevalence. The cut‐off points of UtA PI at >95th percentile and MCA PI and CPR at <5th percentile commonly used in clinical practice were considered abnormal using reference ranges by Rizzo et al.^26^

We inspected the distributions of continuous variables using the Shapiro–Wilk test, histograms and Kernel density plots, then checked for missing data using the Student’s *t*‐test and the chi‐square test of records with and without complete information, respectively, and by graphical methods. We assumed variables were missing at random and imputed 100 data sets over 20 iterations using multiple imputation via fully conditional specification with the mice package in r.^27^ To ensure congeniality, we included all transformed variables in the imputation model, and imputed them passively. Missing fetal biometry and neonate measurement *z*‐scores and percentiles were imputed via the INTERGROWTH‐21^st^ formulae embedded in the r package hbgd.^28^ We checked the final imputation for completeness and plausibility using density plots, box‐and‐whisker plots, summary statistics and comparison of the observed and imputed data before analysis.

Women’s characteristics were summarised using mean (standard deviation, SD) or median (interquartile range, IQR) values, frequencies and percentages. Using the imputed data, we then examined differences in the women’s characteristics in the outcome groups using a two‐sample Student’s *t*‐test, pooled chi‐square statistics, based on Rubin’s procedure,^29^ and pooled estimates of the binomial proportions, based on Wilson’s confidence interval method.^30^ We corrected Pearson’s chi‐square *p*‐values by applying Monte Carlo simulations with 2000 replicates. Significant differences between the pooled estimates of the binomial proportions were assessed using Newcombe’s confidence interval method.^31^ We conducted univariable and multivariable binary logistic regression analyses on the 100 multiply imputed data sets and pooled estimates using Rubin’s rules^29^ to identify the Doppler indices significantly associated with the primary outcomes. Variables for multivariable analysis were selected based on either a univariate analysis *p*‐value of ≤0.27 or clinical importance. We included parity and malaria based on clinical importance. The multivariable model was fitted assuming a two‐sided significance level of 0.05. We checked for confounding by examining the significant variable for a difference in the crude and adjusted coefficient estimate of 15% or more. SA carried out the analysis in r 4.0.4 (15 February 2021).

### 2.7 Ethics statement

We obtained ethical clearance from Makerere University School of Medicine Research and Ethics Committee (ref. 2018–090) and Uganda National Council for Science and Technology (ref. HS 2459), and permission to implement the Ending Preventable Stillbirths by Improving Diagnosis of Babies at Risk (EPID) study in Kagadi territory from both the district and the hospital authorities. All participants were counselled and provided written informed consent. Illiterate participants used a thumbprint. Patients were not involved in the development of this study.

## 3 RESULTS

### 3.1 Participant characteristics

This study was reported in accordance with the Strengthening the Reporting of Observational Studies in Epidemiology (STROBE) statement for cohort studies. We enrolled 1239 pregnant women, 175 of whom were enrolled between 9 ^+ 0^ and 13 ^+ 6^ weeks of gestation. We lost 216 women to follow‐up, mostly as a result of the COVID‐19 lockdown and a temporary breakdown of the ultrasound equipment. Moreover, 22 women had miscarriages, five women gave birth before 33 ^+ 0^ weeks of gestation and one woman had a Doppler scan before 32 weeks of gestation, leaving 995 women for analysis (Figure S1). The cohort characteristics and the extent of missing values are reported in Table S1. The mean gestational age at Doppler scan and at birth was 36.9 weeks (SD, 1.02 weeks) and 39.8 weeks (SD, 1.39 weeks), respectively, whereas the median gestational age at pregnancy dating was 18.4 weeks (range, 9–23 weeks). The median maternal age was 25 years (IQR, 22–30 years), and 188 (18.9%) of the women were nulliparous. Most women (88.9%, *n* = 885) gave birth in a health facility, whereas the remainder gave birth with traditional birth attendants, at home or on their way to hospital. The incidences of stillbirth, neonatal death and CAPO were 1.8% (*n* = 18), 1.3% (*n* = 13) and 13.7% (*n* = 136), respectively. Follow‐up fetal biometry and Doppler scans were obtained from 544 mothers. Birthweight was missing in 13.8% of the neonates, whereas the right uterine artery PI had a high proportion (55.8%) of missing values.

### 3.2 Prevalence of abnormal doppler

The prevalence of UA PI and UtA PI >95th percentile was 4.4% (95% CI 3.2–6.1%) and 5.6% (95% CI 4.3–7.4%), respectively. About 16.4% (95% CI 13.9–19.2%) of the MCA PI and 10.4% (95% CI 8.4–12.8%) of the CPR values were <5th percentile. One pregnancy had absent end‐diastolic flow in the UA.

### 3.3 Predictors of composite adverse perinatal outcome (CAPO)

We compared the characteristics of pregnancies with and without adverse perinatal outcomes (Table 1). The proportion of women with MCA PI and CPR <5th percentile or UtA PI >95th percentile was significantly higher in women who experienced adverse outcomes than in women who did not experience adverse outcomes. In multivariable logistic regression analysis, MCA PI <5th percentile (OR 2.08, 95% CI 1.17–3.70, *p* = 0.013), CPR <5th percentile (OR 2.22, 95% CI 1.13–4.37, *p* = 0.020) and UtA PI >95th percentile were significantly associated with CAPO (Table 2). On the other hand, CPR <5th percentile was strongly associated with stillbirth (OR 4.82, 95% CI 1.09–21.30, *p* = 0.038) (Table 3), but had a weak relationship with perinatal death (Table S4). In contrast, the UA PI >95th percentile had no significant relationships with CAPO, perinatal death or stillbirth. Other important risk factors for adverse perinatal outcomes were syphilis, fetal sex, gestational age at birth and previous stillbirth.

**TABLE 1:** Maternal and pregnancy characteristics in pregnancies with adverse perinatal outcome compared with pregnancies that did not experience an adverse outcome

Characteristic	No adverse perinatal outcome group (*n* = 859)	Adverse perinatal outcome group (*n* = 136)	*p*
Body mass index (kg/m^2^), mean (SD)	24.5 (3.89)	25.1 (4.12)	**0.145**
Log. age, mean (SD)	3.23 (0.24)	3.24 (0.24)	0.636
Nulliparous, yes, %	24.2	28.3	0.367
Malaria, yes, %	40.4	37.6	0.597
Syphilis, yes, %	6.8	12.8	**0.040** *
Previous stillbirth, yes, %	2.4	4.6	0.197
Chronic hypertension, yes, %	1.5	5.3	0.084
Alcohol use in pregnancy, yes, %	14.9	12.2	0.440
Urinary tract infection, yes, %	15.4	19.8	0.259
HIV, positive, %	9.9	12.5	0.437
Sex of baby, male, %	46.8	58.8	**0.009** *
EFW *z*‐score, mean (SD)	0.07 (1.45)	0.02 (1.48)	0.660
AC *z*‐score, mean (SD)	0.06 (1.63)	−0.10 (1.56)	0.212
UA PI >95th percentile, %	4.2	5.4	0.598
UtA PI >95th percentile, %	4.6	11.5	**0.017** *
MCA PI <5th percentile, %	14.8	26.2	**0.014** *
CPR PI <5th percentile, %	9.1	18.2	**0.022** *
Gestational age at Doppler (weeks), mean (SD)	36.8 (1.41)	36.8 (1.34)	0.874
GA at birth, full term	63.9	47.1	**0.001** *
Preterm	3.1	10.3	
Early term	15.1	17.6	
Late term	14.6	19.9	
Post‐term	3.3	5.1	

*Note:* Percentages are pooled proportions estimated from Wilson’s confidence interval method: *N* = 995; *m* = 100 imputed data sets.

* Significant (in bold) at *p* < 0.05; *p*‐value from multiple imputation two‐sample Student’s *t*‐test and pooling chi‐square statistics using Rubin’s procedure; SD, pooled standard deviation; GA, gestational age at birth: preterm, <37 weeks; early term, 37–38 weeks; full term, 39–40 weeks; late term, 41 weeks; post‐term, ≥42 weeks.

**TABLE 2:** Univariable and multivariable logistic regression analysis of composite adverse perinatal outcome prediction from the maternal and pregnancy characteristics in women undergoing routine antenatal care

Characteristic	Univariate		Multivariate			
			Model A		Model B	
	Crude OR (95% CI)	*p*	Adjusted OR (95% CI)	*p*	Adjusted OR (95% CI)	*p*
Body mass index (kg/m^2^)	1.03 (0.98–1.08)	0.145	1.04 (0.99–1.09)	0.082	1.05 (1.00–1.10)	0.051
Syphilis, yes	1.98 (1.04–3.79)	0.037	2.12 (1.05–4.30)	0.035*	2.07 (1.02–4.18)	0.042*
Previous stillbirth, yes	1.93 (0.71–5.26)	0.196	2.13 (0.73–6.22)	0.162	2.29 (0.79–6.62)	0.123
Chronic hypertension, yes	3.51 (0.93–13.27)	0.063	2.89 (0.69–12.11)	0.145	2.74 (0.65–11.58)	0.170
Sex of baby, male	1.62 (1.12–2.34)	0.009	1.66 (1.12–2.47)	0.011*	1.69 (1.14–2.50)	0.009*
AC *z*‐score	0.86 (0.68–1.08)	0.214	0.83 (0.66–1.05)	0.128	0.84 (0.66–1.07)	0.155
Nulliparous, yes	1.23 (0.78–1.93)	0.368	1.31 (0.81–2.13)	0.262	1.30 (0.80–2.11)	0.284
Malaria, yes	0.88 (0.58–1.34)	0.581	0.80 (0.51–1.25)	0.334	0.80 (0.51–1.26)	0.336
GA at birth, full term	Ref.		Ref.		Ref.	
Preterm	4.45 (2.21–8.92)	0.001	4.98 (2.26–10.99)	0.001*	4.84 (2.20–10.66)	0.001*
Early term	1.58 (0.95–2.63)	0.075	1.43 (0.84–2.45)	0.184	1.47 (0.86–2.51)	0.159
Late term	1.85 (1.13–3.03)	0.013	1.87 (1.11–3.14)	0.018*	1.87 (1.11–3.13)	0.017*
Post‐term	2.14 (0.90–5.11)	0.085	2.22 (0.89–5.49)	0.085	2.09 (0.84–5.19)	0.112
UtA PI >95th percentile	2.64 (1.21–5.70)	0.014	2.36 (1.03–5.39)	0.041*	2.39 (1.05–5.44)	0.038*
MCA PI <5th percentile	2.04 (1.17–3.54)	0.012	2.08 (1.17–3.70)	0.013*		
CPR PI <5th percentile	2.20 (1.15–4.20)	0.017			2.22 (1.13–4.37)	0.020*

* Significant at *p* < 0.05; OR, odds ratio after pooling estimates using Rubin’s rule; *N* = 995; *m* = 100 imputed data sets; model A includes MCA PI; model B includes CPR; GA, gestational age at birth: preterm, <37 weeks; early term, 37–38 weeks; full term, 39–40 weeks; late term, 41 weeks; post‐term, ≥42 weeks.

**TABLE 3:** Univariable and multivariable logistic regression analysis of stillbirth prediction from the maternal and pregnancy characteristics in women undergoing routine antenatal care

Characteristic	Univariate		Multivariate			
			Model A		Model B	
	Crude OR (95% CI)	*p*	Adjusted OR (95% CI)	*p*	Adjusted OR (95% CI)	*p*
Body mass index (kg/m^2^)	1.06 (0.96–1.18)	0.231	1.05 (0.95–1.17)	0.336	1.07 (0.96–1.19)	0.197
Syphilis, yes	2.61 (0.62–10.9)	0.188	2.56 (0.57–11.64)	0.221	2.40 (0.52–11.14)	0.263
Previous stillbirth, yes	5.42 (1.15–25.6)	0.032	5.58 (1.06–29.27)	0.042*	6.98 (1.28–37.91)	0.024*
GA at birth, full term	Ref.		Ref.		Ref.	
Preterm	4.44 (0.89–22.13)	0.068	5.25 (0.96–28.81)	0.056	5.06 (0.90–28.51)	0.065
Early term	2.31 (0.67–8.00)	0.186	2.00 (0.51–6.98)	0.335	2.00 (0.53–7.45)	0.303
Late term	1.74 (0.44–6.83)	0.424	1.78 (0.44–7.22)	0.415	1.76 (0.43–7.21)	0.433
Post‐term	5.25 (1.05–26.30)	0.043	6.02 (1.14–31.91)	0.034*	5.57 (1.04–29.76)	0.044*
MCA PI <5th percentile	2.76 (0.60–12.79)	0.193	2.75 (0.56–13.52)	0.211		
CPR PI <5th percentile	4.28 (1.07–17.14)	0.040			4.82 (1.09–21.30)	0.038*

* Significant at *p* < 0.05; OR, odds ratio after pooling estimates using Rubin’s rule; *N* = 995; m = 100 imputed data sets; model A includes MCA PI; model B includes CPR; GA, gestational age at birth: preterm, <37 weeks; early term, 37–38 weeks; full term, 39–40 weeks; late term, 41 weeks; post‐term, ≥42 weeks.

### 3.4 Subgroup analysis in pregnancies with and without SGA neonates

**FIGURE 1:**
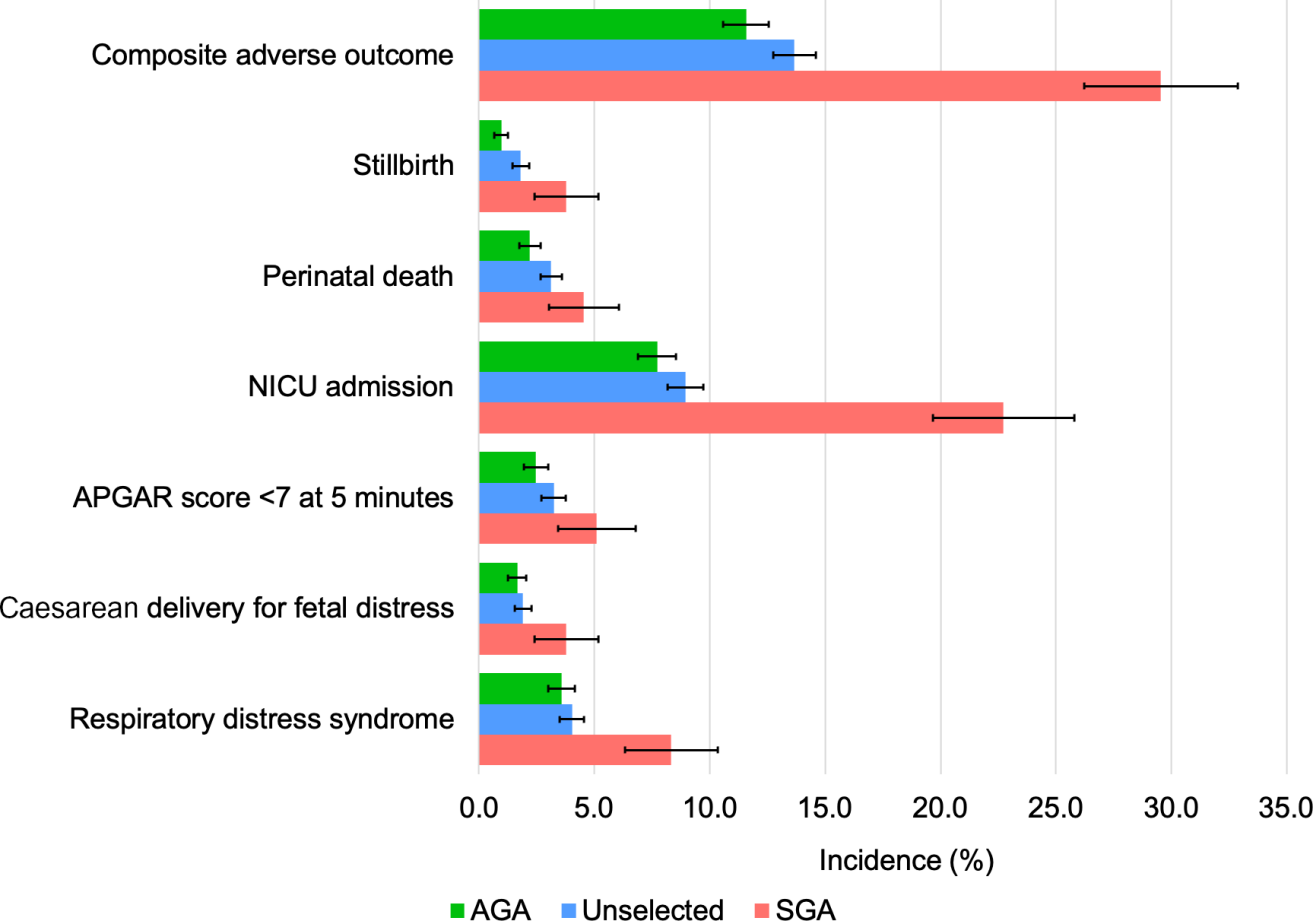
Comparison of the incidence of adverse perinatal outcomes in AGA pregnancies (birthweight ≥10th percentile), in women undergoing routine antenatal care and in SGA pregnancies (birthweight <10th percentile) at Kagadi Hospital, Uganda

The incidence of SGA at birth was 15.4% (95% CI 13.5–17.5%). Adverse perinatal outcome rates were remarkably higher in SGA births than in non‐SGA births (Figure 1). For instance, the incidence of CAPO was 25% (95% CI 23.0–36.1%) in SGA newborns compared with 11.5% (95% CI 9.6–13.5%) in AGA births.

In the SGA subgroup, the proportion of MCA PI <5th percentile was 21.2% (95% CI 14.8–29.5%), whereas the proportion of CPR <5th percentile was 13.9% (95% CI 8.8–21.2%). The proportions of UA PI and UtA PI >95th percentiles were low, about 6.2% (95% CI 3.2–11.5%) and 6.5% (95% CI 3.4–12.4%), respectively. In multivariable logistic regression analysis, MCA PI <5th percentile was associated with CAPO (OR 3.75, 95% CI 1.18–11.88, *p* = 0.025) (Table S2).

Gestational age at birth, fetal sex, chronic hypertension and body mass index were also associated with CAPO in multivariable analysis (Table S3).

## 4 DISCUSSION

### 4.1 Main findings

The proportion of abnormal MCA PI and CPR was quite high, whereas abnormal UA PI was rare. The occurrence of CPR <5th percentile was strongly associated with stillbirth, whereas MCA PI and CPR <5th percentile and UtA PI >95th percentile were independently associated with CAPO. In the SGA subgroup, the association between MCA PI <5th percentile and CAPO was stronger than in women undergoing routine ANC. The incidence of adverse perinatal outcomes was remarkably higher in SGA pregnancies than in AGA pregnancies.

### 4.2 Strengths and limitations

This is one of the few studies with robust methodology from an LMIC setting to investigate the relationship between antenatal Doppler ultrasound in the third trimester and adverse perinatal outcomes. We blinded the Doppler ultrasound results from the attending clinicians who would have used them to manage patients and alter their prognosis. Many variables known to be associated with adverse pregnancy outcomes were included in the analysis and adjusted for as important confounding factors. The sonographers were well trained and met a predefined quality criterion. We handled missing data using multiple imputation, increasing the precision and validity of our findings.

Although 17.4% (*n* = 216) of the women dropped out, mostly as a result of the COVID‐19 lockdown and a temporary breakdown of the ultrasound equipment, this remains one of the largest Doppler studies from a low‐income setting. We examined the women using two different brands of ultrasound machines. It is, however, unlikely that this introduced significant differences in the findings as we used the same equations and reference ranges for biometry and Doppler computations. This reflects the practice in a realistic clinical setting, facilitating the generalisability of our findings. Only a few women (*n* = 175) had a dating scan in the first trimester, but the study used an alternative optimal dating method for women presenting later than 13 weeks of gestation. As we offered all women a single third‐trimester Doppler scan, placental insufficiency may have occurred after the scan, but few women were examined as early as 32 weeks of gestation. This being a single‐centre primary study, caution is urged when extrapolating the findings to other clinical settings.

### 4.3 Interpretation

There is limited evidence on the clinical role of antenatal Doppler in low‐resource settings, where the burden of stillbirths is highest. In this study, abnormal UA PI was rare. The results are comparable with the prevalence of AEDF or REDF (0%–2.13%) reported in low‐risk pregnancies in HICs.^17^ Interestingly, a study from South Africa reported a prevalence of abnormal UA resistive index (RI) (and AREDF) in low‐risk pregnancies of 13.0% (1.2%).^32^ The large difference with our observations could be explained by the classical differences in the pathophysiology of early and late FGR: Hlongwane et al.^32^ enrolled women at 28–34 weeks of gestation, a period in which abnormal UA commonly manifests.

In our study, the proportion of abnormal MCA PI and CPR was quite high, but studies for comparisons were limited. The MCA blood flow redistribution was associated with a twice increased risk of CAPO. Similar results were reported in an HIC study.^15^

Studies from HICs indicate that CPR may be a useful indicator for intrauterine fetal compromise.^33^ Its predictive value for perinatal death was high (with an area under the summary receiver operating characteristic curve (AUC) of 0.83, 95% CI 0.74–0.92),^33^ and low for CAPO (AUC 0.71–0.74).^33^ Similarly, our study found that fetuses with CPR <5th percentile were four times more likely to experience stillbirths and twice as likely to experience CAPO, compared with fetuses with CPR ≥5th percentile. However, this only had borderline significance for perinatal death (*p* = 0.069). Some neonatal deaths may have resulted from causes unrelated to pregnancy complications, like FGR caused by placental insufficiency.

The UtA PI had a significant relationship with CAPO in women undergoing routine ANC. Among the SGA subgroup, the cases were too few (*n* = 153) to allow us to reliably study it, although studies from HICs suggest its potential clinical role in suspected SGA pregnancies in late gestation.^34^

Subgroup analysis in SGA pregnancies showed a low prevalence of UA PI >95th percentile. Studies from HICs indicate that less than 10% of UA PI may be abnormal in SGA pregnancies in late gestation.^5^, ^6^, ^7^, ^8^ In contrast, of the SGA fetuses at >35 weeks of gestation with normal UA, 16 (34%) had MCA redistribution.^7^ In another study involving 171 SGA fetuses examined after 36 weeks of gestation, the proportion of abnormal UA PI was significantly lower than that of abnormal MCA PI (2.9 versus 13.5%; *p* < 0.01) and CPR (2.9 versus 22.8%; *p* < 0.001) before birth,^8^ as observed in our study. In multivariable logistic regression analysis, the risk of CAPO in fetuses with MCA PI <5th percentile was three times as high as that observed in fetuses with MCA PI ≥5th percentile.

Further, as in our study, syphilis, gestational age at birth, fetal sex, previous stillbirth and chronic hypertension are known risk factors for adverse perinatal outcomes.^1^, ^35^ We have enhanced our existing knowledge on the key predictors used for developing risk prediction models for adverse perinatal outcomes in LMICs.^35^

Our study affirms that fetal cerebral blood flow redistribution is an indicator for intrauterine fetal compromise in the third trimester, a finding particularly important in LMICs, where rigorous evidence on the role of Doppler ultrasound in preventing stillbirths is acutely lacking even though LMICs bear the highest burden of disease.^9^, ^36^ Stillbirth rates in sub‐Saharan African settings remain substantially high and interventions to reduce their occurrence need to be prioritised.^37^ In the current study, the adverse perinatal outcome rates were higher in SGA neonates than in non‐SGA neonates, in keeping with previous studies from HICs.^2^, ^3^, ^4^ Prenatal screening for suspected SGA fetuses in whom Doppler ultrasound is thought to be more beneficial seems reasonable.^38^, ^39^ In women undergoing routine ANC, stillbirths were strongly associated with low CPR, indicating that this could help identify fetuses at high risk of dying who require close clinical attention. Many stillbirths in our cohort were attributed to the poor monitoring of mothers at high risk and delayed access to caesarean section delivery. The adoption of practice guidelines can greatly improve the quality of care during pregnancy and reduce the rate of stillbirths. Obstetric guidelines help to ensure that women are put on the appropriate care pathway. However, as in many low‐resource settings, there are no local guidelines for the screening and management of suspected growth‐restricted fetuses in the study setting. Thus, it is important that Doppler ultrasound is embedded into LMICs for use in recommended obstetric populations with context‐tailored evidence‐informed clinical guidelines.^40^ In addition, clinicians must be adequately trained in using Doppler and interpreting the results for managing patients to avoid inappropriate interventions. There is a need to use uniform Doppler reference standards to avoid differences in clinical management arising from using one chart rather than another.^41^

## 5 CONCLUSION

In women undergoing routine ANC in the third trimester, abnormal UA PI was uncommon. Fetal cerebral blood flow redistribution was strongly and independently associated with stillbirth and perinatal complications. We recommend further studies examining its predictive performance and trials evaluating its effectiveness in reducing the risk of perinatal morbidity and mortality in low‐ and middle‐income settings.

## DISCLOSURE OF INTERESTS

SA reports grants from Grand Challenges Canada and University Medical Center Utrecht, Utrecht, The Netherlands, and ultrasound equipment support from the University of Oxford, Oxford, United Kingdom. ATP reports research grants from UK, European and USA research councils and charities: the NIHR / HTA, NIHR Oxford Biomedical Research Centre, EPSRC, GCRF, ERC, NIH and Bill and Melinda Gates Foundation; personal fees and support from Capital Medical University, Beijing; Ministry of Health, Cyprus; from GE Healthcare; and Samsung Medison. ATP holds a patent entitled “A system and method are provided to automatically categorize biological and medical images” US10762630B2, and is a senior advisor for Intelligent Ultrasound. The remaining authors have no disclosures. Completed disclosure of interests form available to view online as supporting information.

## AUTHOR CONTRIBUTIONS

SA, MGK, JB, DEG, DZ, ATP, KKG and MJR contributed to the study conception and design. SA and IMK carried out data collection. SA, EAB and ANK conducted ultrasound training and quality control. SA analysed the data and drafted the article, with regular inputs from KKG and MJR. MGK, JB, DEG, DZ and ATP critically reviewed the work for important intellectual content. All authors approved the final version for publication.

## DETAILS OF ETHICS APPROVAL

This study obtained ethical clearance from Makerere University School of Medicine Research and Ethics Committee (SOMREC) (ref. 2018–090) and from the Uganda National Council for Science and Technology (UNCST) (ref. HS 2459). Further, we obtained permission to operate the EPID study in Kagadi territory from both the district and hospital authorities. All participants provided written and informed consent to participate in this study. Participants who were illiterate provided a thumbprint and were interviewed in the local language.

## Supporting information


Figure S1

Table S1

Table S2

Table S3

Table S4

## Data Availability

The data that support the findings of this study are available from the corresponding author upon reasonable request.
