# Supplementary material for: Middle cerebral arterial flow redistribution is an indicator for intrauterine fetal compromise in late pregnancy in low‐resource settings: A prospective cohort study
Source: BJOG. 2022 Feb 24;129(10):1712–20. doi: 10.1111/1471-0528.17115 (PMC9545180; doi:10.1111/1471-0528.17115)
Supplement: Supplementary file 1 — Figure S1 [file BJO-129-1712-s002.docx]

Total number of women screened

(n= 2056)

Not eligible

(n= 817)

Mothers enrolled

(n= 1239)

**Excluded with reasons,**

(n= 244)

- Lost to follow-up mostly due to COVID-19 lockdown (n= 216)
- Miscarriages (n= 22)
- Birth before 33+0 weeks (n= 5)
- Doppler scan before 32 weeks (n= 01)

Mothers included in the analysis

(n= 995)

**Figure S1**
