# Supplementary material for: Middle cerebral arterial flow redistribution is an indicator for intrauterine fetal compromise in late pregnancy in low‐resource settings: A prospective cohort study
Source: BJOG. 2022 Feb 24;129(10):1712–20. doi: 10.1111/1471-0528.17115 (PMC9545180; doi:10.1111/1471-0528.17115)
Supplement: Supplementary file 2 — Table S1 [file BJO-129-1712-s003.docx]

**Table S1:** Maternal and pregnancy characteristics of the study cohort.

| **Characteristic** | **Overall (N= 995)** | **Missing (%)** |
| --- | --- | --- |
| Maternal height (cm), mean (SD) | 157.1 (6.11) | 0.0 |
| Weight (kg), median (IQR) | 60.0 (54.0 – 68.0) | 0.0 |
| Maternal BMI (kg/m^2^), mean (SD) | 24.6 (3.93) | 0.0 |
| Maternal age (years), median (IQR) | 25.0 (22.0 – 30.0) | 24.4 |
| Nulliparous, yes, n (%) | 188 (18.9) | 24.4 |
| Malaria, yes, n (%) | 299 (30.1) | 24.4 |
| Urinary tract infection, yes, n (%) | 113 (11.4) | 24.4 |
| Syphilis, yes, n (%) | 52 (5.2) | 24.4 |
| HIV, positive, n (%) | 69 (6.9) | 24.5 |
| Previous stillbirth, yes, n (%) | 21 (2.1) | 24.4 |
| Chronic hypertension, yes, n (%) | 9 (0.9) | 24.4 |
| Alcohol use in pregnancy, yes, n (%) | 99 (9.9) | 24.4 |
| Smoking, yes, n (%) | 5 (0.5) | 27.2 |
| Stillbirth, yes, n (%) | 18 (1.8) | 0.0 |
| Neonatal death, yes, n (%) | 13 (1.3) | 0.0 |
| NICU admission, yes, n (%) | 89 (8.9) | 0.0 |
| APGAR score <7 at 5 minutes, yes, n (%) | 26 (2.6) | 53.0 |
| EMCS for fetal distress, yes, n (%) | 19 (1.9) | 0.0 |
| Respiratory distress syndrome, yes, n (%) | 40 (4.0) | 0.0 |
| Composite adverse outcome, yes, n (%) | 136 (13.7) | 0.0 |
| Birth weight, mean (SD) | 3.18 (0.46) | 13.8 |
| Birth weight z-scores, mean (SD) | -0.28 (1.07) | 13.8 |
| Birth weight centiles, mean (SD) | 41.66 (29.31) | 13.8 |
| **Mode of birth** |  |  |
| Vaginal birth, n (%) | 866 (87.0) | 0.0 |
| Cesarean birth, n (%) | 129 (13.0) |  |
| **Place of birth** |  |  |
| Health unit, n (%) | 885 (88.9) | 0.0 |
| Traditional birth attendant (TBA), n (%) | 44 (4.4) |  |
| Home, n (%) | 58 (5.8) |  |
| Way to hospital, n (%) | 8 (0.8) |  |
| Sex of baby, male, n (%) | 482 (48.4) | 0.0 |
| GA at dating scan (weeks), median (IQR) | 18.4 (15.7 – 21.0) | 0.0 |
| GA at Doppler (weeks), mean (SD) | 36.90 (1.02) | 45.3 |
| GA at birth (weeks), mean (SD) | 39.80 (1.39) | 0.0 |
| **GA at birth (weeks)** |  |  |
| Preterm, n (%) | 41 (4.1) | 0.0 |
| Early term, n (%) | 154 (15.5) | 0.0 |
| Full term, n (%) | 613 (61.6) | 0.0 |
| Late term, n (%) | 152 (15.3) | 0.0 |
| Postterm, n (%) | 35 (3.5) | 0.0 |
| EFW (g), mean (SD) | 2829.3 (374.7) | 45.3 |
| EFW z scores, mean (SD) | 0.08 (0.94) | 45.3 |
| AC (cm), mean (SD) | 32.19 (1.93) | 45.3 |
| AC z-scores, mean (SD) | 0.04 (0.97) | 45.3 |
| AC percentiles, mean (SD) | 51.57 (28.06) | 45.3 |
| UA PI, median (IQR) | 18.4 (15.7 – 21.0) | 46.1 |
| UA PI z-scores, mean (SD) | -0.23 (1.04) | 46.1 |
| UA PI percentiles, mean (SD) | 42.75 (28.78) | 46.1 |
| MCA PI, mean (SD) | 1.66 (0.30) | 47.4 |
| CPR PI, median (IQR) | 2.0 (1.70 – 2.32) | 48.1 |
| Right UtA PI, median (IQR) | 0.70 (0.60 – 0.81) | 55.8 |
| Left UtA PI, median (IQR) | 0.73 (0.61 – 0.88) | 51.7 |
| UtA PI, median (IQR) | 0.73 (0.63 – 0.84) | 59.5 |

*SD: standard deviation; IQR: interquartile range; BMI: body mass index; EMCS: Emergency cesarean section; EFW: estimated fetal weight; AC: abdominal circumference: UA: umbilical artery; MCA: middle cerebral artery; CPR: cerebroplacental ratio; UtA: uterine artery; UtA: mean uterine artery; GA: gestational age; preterm: <37 weeks; early term: 37-38 weeks; full term: 39-40 weeks; late term: 41 weeks; postterm: ≥42 weeks.
