# Supplementary material for: Middle cerebral arterial flow redistribution is an indicator for intrauterine fetal compromise in late pregnancy in low‐resource settings: A prospective cohort study
Source: BJOG. 2022 Feb 24;129(10):1712–20. doi: 10.1111/1471-0528.17115 (PMC9545180; doi:10.1111/1471-0528.17115)
Supplement: Supplementary file 3 — Table S2 [file BJO-129-1712-s004.docx]

**Table S2:** Univariable and multivariable logistic regression analysis in prediction of composite adverse perinatal outcome from maternal and pregnancy characteristics in a subgroup of SGA births.

| **Characteristic** | **Univariate** | | **Multivariate** | | | |
| --- | --- | --- | --- | --- | --- | --- |
|  |  | | **Model A** |  | **Model B** | |
|  | **Crude OR**  **(95% CI)** | **P-value** | **Adjusted OR (95% CI)** | **P-value** | **Adjusted OR (95% CI)** | **P-value** |
| Syphilis, yes | 3.42 (0.95 – 12.32) | 0.059 | 4.29 (0.95 – 19.45) | 0.058 | 3.69 (0.87 – 15.64) | 0.075 |
| Nulliparous, yes | 1.02 (0.44 – 2.30) | 0.965 | 1.05 (0.40 – 2.75) | 0.918 | 1.07 (0.42 – 2.70) | 0.891 |
| Malaria, yes | 0.59 (0.25 – 1.35) | 0.215 | 0.48 (0.18 – 1.26) | 0.133 | 0.52 (0.20 – 1.33) | 0.172 |
| GA at birth, full-term | Ref. |  | Ref. |  | Ref. |  |
| Preterm | 9.21 (1.01 – 83.86) | 0.048 | 9.59 (0.82 – 112.28) | 0.071 | 8.59 (0.78 – 94.72) | 0.078 |
| Early term | 1.56 (0.51 – 4.83) | 0.432 | 1.15 (0.32 – 4.21) | 0.827 | 1.22 (0.35 – 4.24) | 0.756 |
| Late term | 1.48 (0.60 – 3.62) | 0.392 | 1.39 (0.51 – 3.78) | 0.511 | 1.44 (0.54 – 3.83) | 0.459 |
| Postterm | 2.91 (0.56 -15.13) | 0.202 | 3.37 (0.54 – 20.97) | 0.191 | 3.05 (0.48 – 19.12) | 0.230 |
| MCA PI <5^th^ percentile | 2.96 (1.04 – 8.48) | 0.042 | 3.75 (1.18 – 11.88) | 0.025* |  |  |
| CPR PI <5^th^ percentile | 2.39 (0.70 – 8.14) | 0.159 |  |  | 2.58 (0.69 – 9.61) | 0.154 |

*Significant at p-value <0.05; OR: odds ratio after pooling estimates using Rubin’s rule; N= 995; m= 100 imputed datasets; Model A includes MCA PI; Model B includes CPR; GA: gestational age at birth; preterm: <37 weeks; early term: 37-38 weeks; full term: 39-40 weeks; late term: 41 weeks; postterm: ≥42 weeks.
