# Supplementary material for: Middle cerebral arterial flow redistribution is an indicator for intrauterine fetal compromise in late pregnancy in low‐resource settings: A prospective cohort study
Source: BJOG. 2022 Feb 24;129(10):1712–20. doi: 10.1111/1471-0528.17115 (PMC9545180; doi:10.1111/1471-0528.17115)
Supplement: Supplementary file 4 — Table S3 [file BJO-129-1712-s001.docx]

**Table S3:** Univariable and multivariable logistic regression analysis of composite adverse perinatal outcome prediction from maternal and pregnancy characteristics in a subgroup of AGA births.

| **Characteristic** | **Univariate** | | **Multivariate** | | | |
| --- | --- | --- | --- | --- | --- | --- |
|  |  | | **Model A** |  | **Model B** | |
|  | **Crude OR**  **(95% CI)** | **P-value** | **Adjusted OR (95% CI)** | **P-value** | **Adjusted OR (95% CI)** | **P-value** |
| Body mass index, (kg/m2) | 1.04 (0.99 – 1.10) | 0.080 | 1.05 (0.99 – 1.11) | 0.070 | 1.05 (1.001 – 1.12) | 0.048* |
| Chronic hypertension, yes | 4.73 (1.22 – 18.38) | 0.024 | 4.25 (1.02 – 17.67) | 0.046* | 4.02 (0.96 – 16.8) | 0.056 |
| Sex of baby, male | 1.59 (1.03 – 2.47) | 0.038 | 1.58 (1.00 – 2.49) | 0.052 | 1.59 (1.01 – 2.52) | 0.047* |
| Nulliparous, yes | 1.15 (0.66 – 2.02) | 0.603 | 1.28 (0.71 – 2.30) | 0.410 | 1.27 (0.70 – 2.29) | 0.424 |
| Malaria, yes | 1.02 (0.62 – 1.68) | 0.929 | 1.02 (0.61 – 1.71) | 0.937 | 1.01 (0.60 – 1.70) | 0.955 |
| GA at birth, full-term | Ref. |  | Ref. |  | Ref. |  |
| Preterm | 4.10 (1.82 – 9.22) | 0.001 | 4.27 (1.76 – 10.35) | 0.001* | 4.18 (1.72 – 10.15) | 0.001* |
| Early term | 1.60 (0.89 – 2.88) | 0.112 | 1.56 (0.85 – 2.87) | 0.152 | 1.57 (0.85 – 2.89) | 0.145 |
| Late term | 1.65 (0.88 – 3.09) | 0.117 | 1.66 (0.87 – 3.19) | 0.125 | 1.63 (0.85 – 3.14) | 0.141 |
| Post term | 1.62 (0.50 – 5.20) | 0.411 | 1.65 (0.50 – 5.44) | 0.413 | 1.59 (0.48 – 5.25) | 0.444 |
| UtA PI >95^th^ percentile | 2.79 (1.13 - 6.84) | 0.024 | 2.54 (0.99 – 6.56) | 0.052* | 2.51 (0.97 - 6.49) | 0.057* |
| MCA PI <5^th^ percentile | 1.65 (0.83 - 3.29) | 0.151 | 1.56 (0.76 – 3.21) | 0.228 |  |  |
| CPR PI <5^th^ percentile | 2.01 (0.91 - 4.44) | 0.084 |  |  | 1.90 (0.82, 4.39) | 0.133 |

*Significant at p-value <0.05; OR: odds ratio after pooling estimates using Rubin’s rule; N= 995; m= 100 imputed datasets; Model A includes MCA PI; Model B includes CPR; GA: gestational age at birth; preterm: <37 weeks; early term: 37-38 weeks; full term: 39-40 weeks; late term: 41 weeks; postterm: ≥42 weeks.
