# Supplementary material for: Middle cerebral arterial flow redistribution is an indicator for intrauterine fetal compromise in late pregnancy in low‐resource settings: A prospective cohort study
Source: BJOG. 2022 Feb 24;129(10):1712–20. doi: 10.1111/1471-0528.17115 (PMC9545180; doi:10.1111/1471-0528.17115)
Supplement: Supplementary file 5 — Table S4 [file BJO-129-1712-s005.docx]

**Table S4:** Univariable and multivariable logistic regression analysis of perinatal death prediction from the maternal and pregnancy characteristics in women undergoing routine antenatal care.

| **Characteristic** | **Univariate** | | **Multivariate** | | | |
| --- | --- | --- | --- | --- | --- | --- |
|  |  | | **Model A** |  | **Model B** | |
|  | **Crude OR**  **(95% CI)** | **P-value** | **Adjusted OR (95% CI)** | **P-value** | **Adjusted OR (95% CI)** | **P-value** |
| Syphilis, yes | 2.47 (0.74 – 8.29) | 0.142 | 2.48 (0.69 – 8.94) | 0.165 | 2.34 (0.65 – 8.50) | 0.192 |
| Previous stillbirth, yes | 5.69 (1.57 – 20.59) | 0.008 | 6.33 (1.57 – 25.46) | 0.009* | 7.45 (1.82 – 30.41) | 0.005* |
| Sex of baby, male | 1.71 (0.82 – 3.57) | 0.151 | 1.73 (0.80 – 3.76) | 0.163 | 1.78 (0.81 – 3.88) | 0.148 |
| GA at birth, full-term | Ref. |  | Ref. |  | Ref. |  |
| Preterm | 6.95 (2.32 – 20.84) | 0.001 | 7.68 (2.32 – 25.48) | 0.001* | 7.38 (2.20 – 24.77) | 0.001* |
| Early term | 2.74 (1.10 – 6.94) | 0.030 | 2.41 (0.92 – 6.29) | 0.071 | 2.52 (0.96 – 6.61) | 0.060 |
| Late term | 1.01 (0.28 – 3.62) | 0.989 | 1.03 (0.28 – 3.81) | 0.969 | 1.05 (0.28 – 3.93) | 0.938 |
| Postterm | 4.70 (1.25 – 17.50) | 0.021 | 4.79 (1.21 – 19.01) | 0.025 | 4.58 (1.15 – 18.24) | 0.030* |
| UtA PI >95^th^ percentile | 2.39 (0.49 – 11.54) | 0.274 | 2.19 (0.44 – 10.83) | 0.336 | 2.22 (0.44 - 11.28) | 0.332 |
| MCA PI <5^th^ percentile | 2.08 (0.62 – 6.98) | 0.236 | 2.08 (0.59 – 7.28) | 0.248 |  |  |
| CPR PI <5^th^ percentile | 2.97 (0.90 – 9.78) | 0.072 |  |  | 3.17 (0.91 – 11.09) | 0.070 |

**Significant at p-value <0.05; OR: odds ratio after pooling estimates using Rubin’s rule; N= 995; m= 100 imputed datasets; Model A includes MCA PI; Model B includes CPR; GA: gestational age at birth; preterm: < 37 weeks; early term: 37-38 weeks; full term: 39-40 weeks; late term: 41 weeks; postterm ≥ 42 weeks.
